# Association of home and community-based services and cognitive function of Chinese older adults: social participation as a mediator

**DOI:** 10.1186/s12877-023-04414-y

**Published:** 2023-10-24

**Authors:** Wenyi Lin, Wanxia Yin, Dinghuan Yuan

**Affiliations:** 1 School of Public Administration, Jinan University, Guangzhou, China; 2 Common Prosperity and National Governance Institute, Jinan University, Guangzhou, China

**Keywords:** Community-based service provision, Community care, Social participation, Cognitive function, Older adults, China

## Abstract

**Background:**

This study makes an effort to examine the impact of home and community-based services on maintaining cognitive function and understand the mediating effect of social participation on the relationship of community services and older adults’ cognitive function in China.

**Method:**

The empirical data comes from the Chinese Longitudinal Healthy Longevity Survey (CLHLS). A total of 38,582 (person-time) respondents were gathered for this study. The two-way fixed effects regression model is used to estimate the associations between independent variables, mediating variables and cognitive ability after controlling for socio-demographic, family responsibility, and time variables.

**Result:**

This study has confirmed that participating in daily and social activities is associated with the cognitive functions of Chinese older adults. Social participation can stimulate cognition. Active participation in outdoor activities, doing housework or taking care of kids, daily playing cards or *mah-jong*, reading books and newspapers, watching TV, and listening to the radio can significantly promote the cognitive ability of older adults. In addition, the findings have indicated the importance of community services for older adults. This study shows personal daily care services, legal aid services, health education services, as well as dealing with family and neighborhood disputes have a positive effect on maintaining older adults’ cognitive functions. Meanwhile, the provision of door-to-door medical services hurts their cognitive functions. This study also illustrates that community-based services can increase the level of older adults’ social participation, and then enhance their level of cognitive function.

**Conclusion:**

This study can inform service provision agencies to develop targeted programs to support older adults’ continued engagement.

## Research background

The number of people with dementia is estimated to be 131.5 million worldwide by 2050 [1]. The development trend of Alzheimer’s disease in China is also not optimistic. By the end of 2018, people with Alzheimer’s disease in China accounted for approximately 25 per cent of the total number of people with Alzheimer’s disease in the world, and the average number of new cases per year has reached 300,000 [2]. Alzheimer’s disease has not only become a problem to be solved urgently in the contemporary medical field but also put forward new requirements and challenges to social governance under the background of aging.

Thus, the Chinese government at all levels has made much effort in the last decades to respond to the prevention of cognitive decline among older adults. The Chinese central government launched its first Development Plan of Social Elderly Care Service System in 2011. Since then, China has spent nearly 10 years promoting the development of community-based health management services guided by the policy concept of combining medical and elderly care [3]. Based on the State Council’s Guiding Opinions on Promoting the Combination of Medical and Elderly Services issued in 2015, community health-based management services for older adults are a vital component of the aforementioned combination. Services including regular physical examinations, out-patient visits, family doctor services, and other community-based care services are provided for older adults over 65 years old, particularly older adults living in the community who are seriously ill, disabled, and partially disabled, as well as those experiencing inconveniences or real difficulties [3]. By the end of 2020, Chinese government has established 970,036 public health service agencies both in urban and rural communities. Particularly, 35,365 community health service centers or stations, 35,762 township hospitals, 608,828 village clinics, and 289,542 outpatient departments have been established [4]. Basic public health service items including disease and emergency prevention services, family health services, family doctor services, and health education services are provided for residents in need. The Chinese central government invested 5 billion RMB in supporting 203 prefectures and cities to carry out home and community-based service pilot programs for older adults from 2016 to 2020 [5].

However, the effects of home and community-based services on delaying nursing home placement of people with cognitive decline remain unclear [6]. Therefore, this study aims to empirically examine the impact of home and community-based services on maintaining cognitive function.

### Literature review

The existing studies have found that individual socio-economic factors, family relationships, family support, community-friendly environment and social service are associated factors with the decline of cognitive function [7]. Various international studies investigate the effects of social participation and community-based services on cognitive function. The decline of cognitive functions is closely related to less participation in social activities [8]. Newson confirmed that social activity participation has a significant effect on cognitive functions [9]. Meanwhile, Saczynski et al. found that social activity participation in different age groups has varying effects on cognitive functions. Particularly, social participation in the middle-aged stage (aged 45 and above) has nothing to do with the decline of cognitive function in the aged population, and the probability of dementia for people who do not participate in or rarely participate in social activities among older adults is two to three times that of others [10]. In addition, Carlson conducted a study to examine the effect of social activity programs on the improvement of cognitive function among older adults and find that cognitive function of older adults who participate in tutoring primary school students’ homework and participating in volunteer work improve [11]. James et al. studied the relationship between cognitive deterioration and visits to relatives and friends, travel, physical exercise and various activity indicators. They found that the cognitive function deterioration rate of older adults who participate in social activities decreases by 47% [12].

Moreover, the effect of social participation on cognitive function in Chinese societies is also examined empirically. Cheng and Chan conducted a study among 62 elderly people with mild cognitive impairment and found that playing *mah-jongg* can improve the cognitive function of older adults [13]. Hsu used the data from a longitudinal survey of middle-aged and older people (aged 45 and above) in Taiwan and found that compared with social activities inside the family, participation in social activities outside the family can ameliorate the cognitive function of middle- and old-aged people as compared with social activities inside the family [14]. In recent years, researchers in mainland China use track survey data to examine the relationship between social participation and cognitive function. Xue used the data from the 2008 and 2012 China Health and Retirement Longitudinal Survey (CHARLS) to examine the association of social participation and cognitive function among the people aged 45 and above in China [15]. Herein, social participation includes the following: interacting with friends; playing *mah-jongg*, chess or cards; attending activities in the community club; going to a sport, social or other kinds of clubs; taking part in a community-related organization; attending an educational or training course, doing volunteer work (or charity work); providing help to family, friends or neighbors who do not live with and pay the respondent for the help; providing care services for a sick or disabled adult; and investing on stocks and using the Internet. The findings indicate that higher level of social participation can improve the cognitive function of older people. Moreover, social participation has a larger effect on males than on females, rural older adults than urban older ones and those 65 and above than those below 65. Different types of social participation have varying degrees of influence on the cognitive function of older people. Particularly, taking part in a community-related organization; interacting with friends; playing *mah-jongg*, chess or cards; and attending activities in the community club, and going to a sport, social or other kinds of clubs have a significantly positive effect on improving cognitive function of middle and old-aged people (aged 45 and above). However, volunteer/charity activities did not significantly affect the cognitive function of middle- and old-aged people. The effect of other types of social participation is also not significant. Possibly because participating in social activities that require cognitive functions (e.g., club activities, card playing, chess, and other intellectual activities) has a stronger stimulating effect on the brain and, thus, has a stronger protective effect on cognitive functions. Meanwhile, activities that do not require high cognitive function (e.g., taking care of the sick and the disabled, dancing, and other physical activities) have weaker protective effects on cognitive function [15].

Zhu and Zeng use the data collected from CHARLS in 2011, 2013 and 2015 to examine the effect of participating in social activities on the cognition of rural older adults in China [16]. Results show that participating in social activities significantly improves the cognition of rural older adults, particularly their episodic memory ability. Furthermore, participation in volunteering, rather than participation in entertainment and educational activities, has a greater effect on improving cognitive ability among rural middle- and old-aged people. The effect of volunteering participation is different from the finding of Xue’s study [15]. Possibly because Xue and Zhu and Zeng used CHARLS data in different periods, thus the time variable has an effect. In addition, Zhu and Zeng found that participation in social activities has a greater effect on the cognitive ability of females, relatively young, highly educated, and working rural middle- and old-aged people [15]. In addition, Zhu and Zeng find participation in social activities has a greater effect on the cognitive ability of females, relatively young, highly educated and working rural middle-aged and older people (aged 45 and above) [16].

Thus, providing family support and community service can help old people with dementia delay institutionalization. However, there is a high burden of family caregivers who care for older adults with dementia [17]. In addition, it is difficult for older adults with dementia to get access to community-based agencies which can support their functional abilities and maintain social participation [18, 19]. Vecchio et al. analyzed the association between cognitive impairment and community service use patterns among 59,352 home-care older people aged 65 and over from 2007–2008 [17]. They found that more access to respite care, daycare centre, counselling service and care coordination but less access to domestic assistance and home maintenance are associated with cognitive impairment. Moreover, cognitive impairment is associated with accessing more hours of informal care, and day care centre can reduce caregivers’ stress, depression, and anger [17].

Yu et al. used the 2014 Chinese Longitudinal Healthy Longevity Survey (CLHLS) dataset to explore the relationship between community support service and cognitive function among 6,154 older adults in China [20]. Results show that community support service is a protective factor of cognitive function among older adults in China. However, this study does not analyze the effect of specific service items on cognitive function, therefore, it cannot provide practical implications on what kind of community service is helpful to delay cognitive impairment.

Bai used the data from the 2005, 2008,2011, and 2014 CLHLS to investigate the supply and effect of elderly health management services in urban and rural communities of China [3]. The findings indicate that providing spiritual comfort services in the community significantly reduces the risk of cognitive impairment in urban and rural older adults, however, the effect is more evident for rural older adults. Furthermore, providing health education in the community significantly reduced the risk of cognitive impairment for urban older adults.

In addition, the relationship between community service provision and social participation has been discussed. Community-provided elderly care services can improve the social participation of older adults [21–23]. Nevertheless, the mediating effect of social participation on the association of community-based services and cognitive function has not been examined.

To summarize, existing studies confirm the protective effect of activity engagement on age-related cognitive decline. In Chinese societies, although some researchers have used panel data to analyze the effect of activity engagement on cognitive function among older adults, the effect varies by different activity engagement item. Empirical studies on the association between community-based service and cognitive function are limited. Thus, this study is important because there is an increasing number of Chinese old people with dementia and they demand more community-based aged services in the future. Moreover, considering that the number of older adults with dementia is greater than those living in institutional settings, it is critical to understand the effect of community-based aged service provision on the changes in cognitive status among older adults. This study can inform service provider agencies to develop targeted programs to support older adults’ continued engagement. Appropriate strategies and programs can also be developed to prevent the decline of cognitive functions.

## Method

### Data source

The survey data of this study come from the CLHLS. CLHLS is conducted by Center for Healthy Aging and Development Studies (See http://chads.nsd.pku.edu.cn/sjzx/index.htm for further details) . Ethical approval was not required for the secondary analysis of data from the CLHLS survey. The survey covers a total of 23 provinces and autonomous regions in China. The older adults aged 65 and above and the adult children aged 35–64 were enrolled in the survey. The first baseline survey was conducted in 1998, hence, the project has since completed seven follow-up visits in 2000, 2002, 2005, 2008–2009, 2011–2012, 2014, and 2017–2018 and continuously added new samples in each survey. In the third follow-up visit since 2005, CLHLS surveyed the community service provision for older adults. Thus, this study analyzed the data sets between 2005 and 2018. In addition, the older adults aged 65 and above engaging in two or more round surveys were selected for analysis. A total of 38,582 (person-time) respondents were gathered for this study. Missing data in the variables of this studyis not included in the analysis. Table 1 shows the distribution of the sample size.

**Table 1:** Sample distribution (2005-2018)

**Cohort**	**2005**	**2008**	**2011**	**2014**	**2018**
**1891-1900**	97	101	18	8	0
**1901-1905**	501	677	287	81	7
**1906-1910**	492	1030	771	296	60
**1911-1915**	949	1384	875	463	133
**1916-1920**	1184	1967	1397	720	196
**1921-1925**	822	1505	1274	901	392
**1926-1930**	1112	1553	1313	1051	549
**1931-1935**	1174	1380	1199	1082	697
**1936-1940**	1138	1403	1249	1118	785
**1941-1945**	5	662	751	707	539
**1946-1950**	0	47	121	146	135
**1951-1954**	0	0	10	35	33
**Total**	7474	11709	9265	6608	3526

### Dependent variable

The dependent variable of this study is the cognitive ability of older adults. The Mini-Mental State Examination (MMSE) is used to measure cognitive ability [24]. The MMSE evaluates the cognitive impairment of older adults from five following aspects: general ability (12 items), reaction ability (three items), attention and calculation ability (six items), memory (three items), and language and coordination ability (six items). A score of 1 is given for every correct answer and 0 for the wrong answer, and the total score is 30. The higher the score, the better the cognitive ability of the older adults. MMSE can be used to screen for Alzheimer’s disease. The international standard is 27–30, 21–26, 10–20, and 0–9 points for normal cognition, mild cognitive impairment, moderate cognitive impairment, and severe cognitive impairment, respectively.

### Independent variable

This study takes social participation and community service providers as independent variables. Social participation is measured by the following eight items: Individual outdoor activities, participating in social activities, playing cards or mahjong, reading books and newspapers, watching TV and listening to the radio, taking care of family situations, raising poultry, planting flowers, and pets (0 = no, 1 = yes). The number of eight daily activities measured the level of daily activities participation. Community service provision is measured by whether or not the respondent’s community had provided the eight types of social services for older adults, namely, personal daily care services, door-to-door medical services, psychological consulting, daily shopping, social entertainment services, legal aid services, health education service, and dealing with family and neighborhood disputes (0 = no, 1 = yes). The total number of community elderly care services is measured by the number of eight social services provided by the community to older adults.

### Control variables

Based on the existing research on the influencing factors of the cognitive function of the elderly, variables including individual characteristics, health behavioral habits, family support status, and social security of older adults were selected as control variables.

Individual characteristics include gender (dummy variable, 0 = female, 1 = male), age (continuous variable generates logarithmic value), place of residence (dummy variable, 0 = rural, 1 = urban), years of education (continuous variable) variable), marital status (dummy variable, 0 = unmarried, 1 = married), economic status, and health variables. In terms of economic status, this study uses “Are all your sources of living enough?” to measure the individual economic status. In addition, this study assigns “enough” as 1, and “not enough” as 0. In terms of health, this study uses “How do you think your own health is now?” to measure the individual health status. “Very good” and “good” were assigned a value of 1, and the other three options were assigned a value of 0, and the respondent’s choice of “unable to answer” is deleted. In terms of mental health, it is measured by the “Personality and Emotional Traits” section in the CLHLS questionnaire, including: (a) Whether you can think well (very good, good, average, hard, and very hard) no matter what you encounter; (b) Do you like to keep things clean and tidy (very like, like, average, dislike, and dislike very much); (c) Do you feel energized (always, often, sometimes, rarely, and never); (d) Do you feel ashamed, regretful, or guilty about what you have done (always, often, sometimes, rarely, and never); (e) Do you feel uncomfortable with people around you (always, often, sometimes, rarely, and never); or (f) Whether your own affairs are in your own hands (always, often, sometimes, rarely, and never); (g) Do you often feel that the people around you are untrustworthy (always, often, sometimes, rarely, and never), and assign the first, second, third, and sixth positive points to 1–5, and the fourth, fifth, seventh negative points to 5–1, and then add up the mental health score. The higher the score, the worse the mental health of older adults and the more negative life attitude. In terms of life satisfaction, this study uses “How do you think your life is now” to measure the individual life satisfaction. If the respondent answered “very good” or “good”, it indicates the respondent is satisfied with life (life satisfaction = 1). In terms of healthy behaviors, this study selected the questions of “whether you smoke often”, “whether you drink alcohol often”, and “whether you often exercise’ to measure healthy behavior habits and assign the values ​​as “0 = no, 1 = yes”, respectively.

In terms of family support status, the two dimensions of emotional support provided by the family and living conditions are considered. First, based on the question of “Do you communicate with your family frequently”, the variable “family provides emotional support” is constructed. The family is considered to have provided emotional support if there is more than one frequent contact among all children, and the value is 1; otherwise, it is 0. Second, the living pattern of older adults with their children may affect the frequency of their interaction with relatives and the availability of informal support, thereby affecting the physical and mental health and cognitive ability of older adults. In this study, the question “Who lives with you” is used to measure the living conditions of the elderly. Residence status is a dummy variable (0 = living alone, 1 = not living alone).

In terms of the variables of social security status, pension, and medical security are included. For old-age security and insurance, older adults who have any kind of old-age security and insurance items are defined as 1, otherwise, they are defined as 0. For medical insurance, older adults who participated in any medical insurance program were defined as 1, otherwise, were defined as 0. In terms of access to health services, information was obtained by asking respondents “If you are seriously ill, can you go to the hospital for treatment in time?”. The answer ‘cannot’ was defined as 0, and ‘can’ was defined as 1.

### Data analysis

The Heckman two-stage model is suitable for solving the endogeneity problem caused by the sample selection bias, hence, the Heckman test was performed, which showed that the sample selection bias was not serious. Afterward, the Hausman test was performed, which showed that a fixed-effect model should be used. Moreover, the two-way fixed effects regression model is used to estimate the associations between independent variables, mediating variables and cognitive ability after controlling for socio-demographic, family responsibility, and time variables. Variables from five waves are used.

## Results

Table 2 shows the descriptive statistics of the sample from 2005 to 2018. The average MMSE score of Chinese older adults was 23.3 points, thereby indicating mild cognitive impairment. As illustrated in the table, the interviewed respondents were older, with an average age of 84.28 years. The number of women exceeded that of men, accounting for 54.4% of the total sample. Most older adults (55.9%) lived in rural areas. However, the number of years of education of older adults was generally low, with an average of only 2.37 years. Most respondents (59.8%) were unmarried, and 83.5% were living alone. Nearly 60% of the respondents said that they were responsible for cooking, taking care of the kids, and other household chores. Only 30% of the respondents said they had pension insurance, and 70% said they had medical insurance. Meanwhile, in terms of access to health care, only 6.4% of the respondents said that they could not go to the hospital for timely treatment when they were seriously ill.

**Table 2:** Sample description

**Variable**	**2005**	**2008**	**2011**	**2014**	**2018**	**Total sample**
Cognitive ability	24.72	22.51	22.74	23.59	23.84	23.3
	(7.211)	(9.045)	(8.824)	(8.246)	(8.081)	(8.48)
Age	81.45	84.54	85.47	84.94	85.03	84.28
	(10.879)	(11.366)	(11.22)	(10.572)	(9.264)	(11.017)
Gender (female) (%)	55.1	55.3	54.5	53.2	51.7	54.4
Area type (rural) (%)	58.7	59.7	50.9	53.9	53.6	55.9
Marital status (unmarried) (%)	57.6	61.8	61.0	58.2	57.0	59.8
Years of education	2.32	2.28	2.34	2.48	2.95	2.37
	(3.609)	(3.547)	(3.532)	(3.517)	(3.841)	(3.565)
Self-assessed health status (good) (%)	53.2	49.8	45.5	44.8	48.0	48.5
Mental health	15.69	16.55	15.58	15.81	21.48	16.43
	(4.12)	(3.987)	(3.997)	(4.032)	(2.147)	(4.211)
Life satisfaction (Satisfactory) (%)	60.0	59.3	61.0	67.2	70.7	62.2
Smoking (no) (%)	77.1	80.6	81.0	81.9	83.1	80.4
Drinking alcohol (no) (%)	77.4	81.3	82.4	83.7	83.8	81.4
Exercise regularly (no) (%)	64.1	67.8	64.0	70.4	70.1	66.8
Lives alone (no) (%)	85.9	84.2	83.2	81.1	81.4	83.5
Frequent correspondence (no) (%)	21.7	13.9	12.3	9.1	9.8	13.9
Does housework or take care of kids (no) (%)	31.0	40.1	43.8	40.4	44.8	39.7
Personal outdoor activities (no) (%)	26.2	35.2	38.8	39.2	32.8	34.8
Reads books and newspapers (no) (%)	75.4	81.1	79.3	78.5	81.9	79.2
Watches TV and listens to the radio (no) (%)	24.5	27.6	30.1	27.2	27.4	27.5
Plays cards or *mahjong* (no) (%)	78.6	83.5	84.1	82.2	85.0	82.6
Plant flowers and keep pets (no) (%)	80.4	84.4	78.5	77.5	82.5	80.8
Raising poultry (no) (%)	64.9	72.1	74.3	72.3	76.7	71.6
Engages in social activities (no) (%)	82.7	87.1	85.7	85.2	88.9	85.7
Personal daily care services (no) (%)	96.4	95.2	95.8	94.9	91.6	95.2
Door-to-door medical service (no) (%)	89.3	91.3	72.3	65.5	68.4	79.7
Psychological consulting (no) (%)	94.1	93.7	93.1	91.3	87.5	92.7
Daily shopping service (no) (%)	94.5	94.7	93.2	89.5	88.4	92.8
Social entertainment services (no) (%)	88.9	89.4	86.0	82.1	80.6	86.4
Legal aid services (no) (%)	92.9	93.7	89.2	86.8	82.3	90.2
Health education services (no) (%)	90.4	91.8	68.4	60.2	61.0	77.5
Dealing with family and neighborhood disputes (no) (%)	80.7	82.7	78.0	74.3	71.0	78.6
Old-aged insurance (yes) (%)	24.3	22.9	33.6	38.1	37.9	/
Medical insurance (yes) (%)	26.0	71.0	85.5	89.9	86.3	/
Access to health care (yes) (%)	89.6	92.7	94.5	96.5	97.6	/

More than 70% of older adults watched TV or listened to the radio, whereas only 20% read books and newspapers daily. Of the elderly, 65.2% participated in Tai Chi, square dance, and other outdoor activities, whereas 85.7% said they did not participate in social activities, and 78.6% did not play cards or *mah-jong*.

The most community services provided for older adults were health education services (accounting for approximately 22.5%). Of the elderly, 95.2% said that personal daily care services were not provided in their communities. Nevertheless, the proportion of community services provided for older adults was increasing annually, among which the largest increase was the supply of health education services.

Table 3 illustrates the association between social participation and older adults’ cognitive ability. Model 2 in Table 3 shows that the higher the level of social participation, the higher the cognitive ability score of older adults (B = 0.567, *P* < 0.01). For each unit increase in the level of social engagement, the cognitive ability MMSE score increased by 0.567 units. Results show that social participation has a positive effect on maintaining or improving the cognitive level of the elderly.

**Table 3:** Association of social participation and cognitive ability

	Model 1			Model 2			Model 3		
	coefficient	t	P> | t |	coefficient	t	P> | t |	coefficient	t	P> | t |
Personal outdoor activities(no=0)	\	\	\	\	\	\	0.946	11.870	0.000
Engages in social activities (no=0)	\	\	\	\	\	\	0.347	4.930	0.000
Plays cards or mahjong (no=0)	\	\	\	\	\	\	0.479	5.890	0.000
Reads books and newspapers (no=0)	\	\	\	\	\	\	0.381	4.510	0.000
Watches TV and listens to the radio(no=0)	\	\	\	\	\	\	1.100	10.320	0.000
Does housework or takes care of kids (no=0)	\	\	\	\	\	\	0.989	11.540	0.000
Raising poultry (no=0)	\	\	\	\	\	\	0.300	3.930	0.000
Plant flowers and keep pets (no=0)	\	\	\	\	\	\	-0.069	-0.990	0.323
Level of social participation	\	\	\	0.567	23.760	0.000	\	\	\
Gender(female=0)	0.771	0.810	0.419	1.182	1.370	0.171	1.230	1.440	0.151
Age(logarithm)	22.042	3.100	0.002	23.970	4.170	0.000	23.508	4.230	0.000
Area type (rural=0)	0.020	0.220	0.823	-0.050	-0.630	0.532	-0.030	-0.370	0.709
Years of education (logarithm)	0.062	0.880	0.380	0.013	0.240	0.813	0.018	0.330	0.745
Marital status (unmarried=0)	0.262	1.840	0.066	0.241	1.910	0.056	0.233	1.850	0.064
Self-assessed health status(poor=0)	0.522	6.690	0.000	0.435	6.330	0.000	0.407	5.940	0.000
Mental health	-0.126	-11.260	0.000	-0.109	-11.340	0.000	-0.112	-11.730	0.000
Life satisfaction (unsatisfactory =0)	0.197	2.380	0.017	0.177	2.440	0.015	0.218	3.020	0.002
Smoking (no=0)	0.238	1.680	0.093	0.109	0.900	0.370	0.121	1.000	0.316
Drinking alcohol (no=0)	0.092	0.800	0.422	0.043	0.420	0.675	0.056	0.560	0.578
Exercise regularly (no=0)	0.599	7.780	0.000	0.166	2.420	0.016	0.139	2.010	0.045
Living alone (yes=0)	-0.461	-3.500	0.000	-0.452	-3.870	0.000	-0.368	-3.160	0.002
Frequent correspondence (no=0)	0.508	3.270	0.001	0.342	2.680	0.007	0.396	3.110	0.002
Old-aged insurance (no=0)	0.176	0.091	1.940	0.165	1.810	0.070	0.052	-0.002	0.354
Medical insurance (no=0)	0.465	0.082	5.690	0.484	5.890	0.000	0.000	0.305	0.626
Access to health care service (no=0)	0.592	0.155	3.810	0.605	3.880	0.000	0.000	0.287	0.896
Year									
2008	-1.994	-6.630	0.000	-2.022	-8.21	0.000	-1.792	-7.570	0.000
2011	-3.546	-6.110	0.000	-3.643	-7.71	0.000	-3.214	-7.020	0.000
2014	-4.589	-5.560	0.000	-4.756	-7.10	0.000	-4.262	-6.570	0.000
2018	-5.777	-4.870	0.000	-5.764	-5.97	0.000	-5.283	-5.650	0.000
Coefficient	-68.791	-2.220	0.026	-79.533	-3.18	0.001	-77.586	-3.210	0.001
rho	0.7603			rho	0.7449		rho	0.739	

Model 3 in Table 3 shows the cognitive ability of the elderly who actively participate in outdoor activities (B = 0.946, *P* < 0.01) or social activities provide by community service organizations (B = 0.347, *P* < 0.05) and play cards or *mah-jong* (B = 0.479, *P* < 0.01) was better than that of older adults who did not participate in the above activities, thereby indicating that social participation has a certain degree of protection on the cognitive ability of older adults. *Mah-jong* is a kind of traditional game in China and it can stimulate the brain of older adults. In addition, older adults can contact with other players when playing the game, thus the game has social attributes. Similarly, reading books and newspapers (B = 0.381, *P* < 0.001), watching TV, and listening to the radio (B = 1.100, *P* < 0.01) had a significant impact on the cognitive level of older adults. In terms of household chores, the cognitive ability of older adults who need to take care of the family is better than that of the elderly who do not need to do housework (B = 0.989, *P* < 0.001). Nevertheless, it may also be due to the endogeneity problem of reverse causality that the elderly with mild or no cognitive impairment can normally participate in housework activities. Similar results were observed for poultry-raising activities, with older adults reporting higher levels of cognition (B = 0.300, *P* < 0.001).

Table 4 reports the association between community service provision and cognitive ability among older adults. The cognitive abilities of older adults who lived in the community that provides legal aid services (B = 0.213, *P* < 0.01), health education services (B = 0.209, *P* < 0.05), and family-neighborhood dispute handling services (B = 0.331, *P* < 0.001) were higher than those of older adults in their community wherein these services were not provided. However, the cognitive level of older adults who reported that the community provided door-to-door medical services (B = -0.162, *P* < 0.1) was weaker than that of older adults who did not provide this service in the community. In addition, the cognitive level of older adults with old-age insurance was significantly higher than that of the group without old-age insurance (B = 0.20, *P* < 0.05). Moreover, medical insurance had a significant positive effect on the cognitive ability of older adults, and the cognitive ability score of the elderly with medical insurance was higher (B = 0.40, *P* < 0.001). Similarly, access to medical services had a significant positive effect on the cognitive ability of older adults, and the cognitive level of the elderly group who reported that they can go to the hospital in time when they are seriously ill was higher (B = 0.54, *P* < 0.001).

**Table 4:** Association of community services and cognitive ability

	**Model 4**			**Model 5**			**Model 6**		
	**coefficient**	**t**	**P>|t|**	**coefficient**	**t**	**P>|t|**	**coefficient**	**t**	**P>|t|**
Personal daily care services(no=0)	\	\	\	\	\	\	0.34	1.87	0.06
Door-to-door medical service (no=0)	\	\	\	\	\	\	-0.18	-1.96	0.05
Psychological consulting (no=0)	\	\	\	\	\	\	0.21	1.61	0.11
Daily shopping service(no=0)	\	\	\	\	\	\	0.12	0.91	0.36
Social entertainment services(no=0)	\	\	\	\	\	\	0.02	0.24	0.81
Legal aid(no=0)	\	\	\	\	\	\	0.21	1.75	0.08
Health education services(no=0)	\	\	\	\	\	\	0.19	1.99	0.05
Dealing with family and neighborhood disputes (no=0)	\	\	\	\	\	\	0.32	3.88	0.00
Personal outdoor activities(no=0)	\	\	\	0.95	11.87	0.00	0.88	10.76	0.00
Engages in social activities (no=0)	\	\	\	0.35	4.93	0.00	0.28	3.71	0.00
Plays cards or *mah-jong*(no=0)	\	\	\	0.48	5.89	0.00	0.50	6.02	0.00
Reads books and newspapers (no=0)	\	\	\	0.38	4.51	0.00	0.34	3.81	0.00
Watches TV and listens to the radio(no=0)	\	\	\	1.10	10.32	0.00	1.04	9.46	0.00
Does housework or take care of kids (no=0)	\	\	\	0.99	11.54	0.00	0.95	10.82	0.00
Raising poultry(no=0)	\	\	\	0.30	3.93	0.00	0.28	3.59	0.00
Plant flowers and keep pets(no=0)	\	\	\	-0.07	-0.99	0.32	-0.04	-0.60	0.55
Level of social participation	0.77	0.81	0.42	1.23	1.44	0.15	1.83	1.99	0.05
Gender(female=0)	22.04	3.10	0.00	23.51	4.23	0.00	22.87	4.07	0.00
Age(logarithm)	0.02	0.22	0.82	-0.03	-0.37	0.71	-0.01	-0.16	0.87
Area type (rural=0)	0.06	0.88	0.38	0.02	0.33	0.75	0.02	0.27	0.79
Years of education (logarithm)	0.26	1.84	0.07	0.23	1.85	0.06	0.25	1.91	0.06
Marital status(unmarried=0)	0.52	6.69	0.00	0.41	5.94	0.00	0.43	6.03	0.00
Self-assessed health status(poor=0)	-0.13	-11.26	0.00	-0.11	-11.73	0.00	-0.11	-11.00	0.00
Mental health	0.20	2.38	0.02	0.22	3.02	0.00	0.17	2.34	0.02
Life satisfaction(unsatisfactory =0)	0.24	1.68	0.09	0.12	1.00	0.32	0.18	1.48	0.14
Smoking(no=0)	0.09	0.80	0.42	0.06	0.56	0.58	0.04	0.39	0.69
Drinking alcohol(no=0)	0.60	7.78	0.00	0.14	2.01	0.05	0.12	1.66	0.10
Exercise regularly (no=0)	-0.46	-3.50	0.00	-0.37	-3.16	0.00	-0.39	-3.29	0.00
Living alone(yes=0)	0.51	3.27	0.00	0.40	3.11	0.00	0.35	2.67	0.01
Frequent correspondence(no=0)	0.195	1.630	0.103	0.176	1.940	0.052	0.20	2.16	0.03
Old-aged insurance(no=0)	0.582	5.710	0.000	0.465	5.690	0.000	0.40	4.69	0.00
Medical insurance(no=0)	0.607	3.100	0.002	0.592	3.810	0.000	0.54	3.33	0.00
Year									
2008	-1.99	-6.63	0.00	-1.79	-7.57	0.00	-1.96	-8.03	0.00
2011	-3.55	-6.11	0.00	-3.21	-7.02	0.00	-3.41	-7.33	0.00
2014	-4.59	-5.56	0.00	-4.26	-6.57	0.00	-4.55	-6.90	0.00
2018	-5.78	-4.87	0.00	-5.28	-5.65	0.00	-5.58	-5.88	0.00
Coefficient	-68.79	-2.22	0.03	-77.59	-3.21	0.00	-75.73	-3.10	0.00
rho	0.760		rho	0.739	rho	0.735	rho	0.760	

Table 5 presents the mediating effect of social participation on the relationship between community service provision and cognitive ability. Results showed that community services had a significant positive effect on the level of social participation of older adults (B = 0.107, *P* < 0.001). When the level of social participation is included, the direct effect of the number of community services on the cognitive ability of older adults remains significant (B = 0.149, *P* < 0.001). The level of social participation also has a direct positive and significant effect on the cognitive ability of older adults. The more social participation, the higher the cognitive score of older adults (B = 0.545, *P* < 0.001).

**Table 5:** The mediating effect of social participation on the relationship between community service and cognitive ability

**DVIVs**	**DV: Cognitive ability**			**DV: Level of social participation**			**DV: Cognitive ability**		
	**coefficient**	**t**	**P > | t |**	**coefficient**	**t**	**P > | t |**	**coefficient**	**t**	**P > | t |**
Number of community service provision	0.206	10.580	0.000	0.107	16.190	0.000	0.149	7.670	0.000
Social participation	/	/	/	/	/	/	0.545	21.900	0.000
Gender(female=0)	2.124	2.250	0.024	0.342	1.260	0.208	1.934	2.080	0.037
Age(logarithm)	27.401	4.000	0.000	5.007	2.390	0.017	24.462	4.080	0.000
Area type (rural=0)	0.280	2.120	0.034	0.104	2.430	0.015	0.237	1.820	0.068
Years of education (logarithm)	-0.012	-0.150	0.880	0.008	0.320	0.753	-0.007	-0.080	0.936
Marital status (unmarried=0)	0.070	1.230	0.220	0.085	3.590	0.000	0.016	0.280	0.780
Self-assessed health status(poor=0)	0.251	3.340	0.001	0.115	5.030	0.000	0.184	2.480	0.013
Mental health	0.235	1.850	0.064	0.118	2.760	0.006	0.181	1.470	0.142
Life satisfaction (unsatisfactory =0)	0.139	1.310	0.190	0.166	4.780	0.000	0.049	0.470	0.637
Smoking (no=0)	0.524	7.410	0.000	0.646	27.330	0.000	0.176	2.500	0.012
Drinking alcohol (no=0)	0.533	7.390	0.000	0.171	7.430	0.000	0.448	6.280	0.000
Exercise regularly (no=0)	-0.125	-12.420	0.000	-0.020	-6.570	0.000	-0.115	-11.640	0.000
Living alone (yes=0)	-0.536	-4.440	0.000	-0.195	-5.520	0.000	-0.437	-3.650	0.000
Frequent correspondence (no=0)	0.474	3.540	0.000	0.118	3.030	0.002	0.398	3.020	0.003
Old-aged insurance (no=0)	0.249	2.64	0.008	0.136	4.25	0.000	0.178	1.91	0.056
Medical insurance (no=0)	0.480	5.54	0.000	0.126	4.77	0.000	0.409	4.79	0.000
Access to health care service (no=0)	0.612	3.71	0.000	0.083	1.86	0.063	0.550	3.40	0.001
Year									
2008	-2.203	-7.660	0.000	-0.661	-7.620	0.000	-1.831	-7.150	0.000
2011	-4.043	-7.260	0.000	-1.114	-6.630	0.000	-3.418	-6.940	0.000
2014	-5.409	-6.830	0.000	-1.408	-5.880	0.000	-4.604	-6.590	0.000
2018	-6.822	-5.960	0.000	-2.082	-6.020	0.000	-5.630	-5.580	0.000
Coefficient	-92.228	-3.100	0.002	-18.347	-2.010	0.044	-81.317	-3.120	0.002
rho		0.779		rho	0.667		rho	0.749	
	F(18,12375) = 76.35			F(18,12373) = 154.5			F(19,12373) = 89.23		
	Prob > F = 0.000			Prob > F = 0.000			Prob > F = 0.000		

Table 6 shows the result of the Sobel test. The Z values of the Sobel test are all significantly positive, which proves that the results of partial mediation are established. That is, community services can improve the cognitive ability of older adults by improving their level of social participation. Providing community service can not only directly affect the cognitive ability of older adults but also indirectly affect the cognitive level through the mediating effect of social participation. The direct effect of community services and the mediating effect through the level of social participation were 0.064 and 0.074, accounting for 46.2% and 53.6% of the total effect (0.139), respectively.

**Table 6:** Sobel test

	**Coefficient**	**SE**	**Z**	**P>|Z|**
Sobel	0.075	0.004	17.110	0.000
Goodman-1(Aroian)	0.075	0.004	17.110	0.000
Goodman-2	0.075	0.004	17.110	0.000
a coefficient	0.097	0.005	18.7734	0.000
b coefficient	0.771	0.019	41.575	0.000
Indirect effect	0.075	0.004	17.110	0.000
Direct effect	0.064	0.017	3.860	0.000
Total effect	0.139	0.017	8.156	0.000
Proportion of total effect that is mediated		0.537		
Ratio of indirect to direct effect		1.160		
Ratio of total to direct effect		2.160		

## Discussion

This study has confirmed that participating in daily activities is associated with the cognitive functions of Chinese older adults. Social participation can stimulate cognition. Active participation in outdoor activities, doing housework or taking care of kids, daily playing cards or *mah-jong*, reading books and newspapers, watching TV, and listening to the radio can significantly promote the cognitive ability of older adults (Table 3). The finding is similar to the findings of Xue [15] as well as Zhu and Zeng [16], and support the international literature [12, 25, 26].

In addition, the findings have indicated the importance of community services for older adults, which have been pointed out by Zhang & Su [27], Wei and Wang [28], and Yu et al. [29]. But Zhang & Su, Wei and Wang, and Yu et al. just examined the importance of the number of community service items, without considering the impacts of specific community items on maintaining their cognitive functions. This study also shows personal daily care services, legal aid services, health education services, as well as dealing with family and neighborhood disputes have a positive effect on maintaining older adults’ cognitive functions. Meanwhile, the provision of door-to-door medical services is negatively associated with their cognitive functions (Table 4). A possible explanation is that if the community provides door-to-door medical services, then older adults do not need to go out of the community, and outdoor activities would be reduced, thus declining their cognitive function. Another possible explanation is that some older people may already be experiencing cognitive decline and may be using door-to-door services.

This study also illustrates that community-based services can increase the level of older adults’ social participation, and then enhance their level of cognitive function (Table 5). On the one hand, community-based services may create more opportunities for older adults to participate in social activities. On the other hand, community services are likely to improve the physical and mental health of older adults and help them maintain the physical and mental conditions they need for later years, thereby indirectly improving their ability to participate in social activities and expanding their social participation rate [16].

The community provides older adults with daily care, health rehabilitation, legal aid, and other elderly care services, which can enable them to receive various services in a familiar living environment, which is important in maintaining their cognition (Table 4). The health education services provided by the community play a preventive role in guiding the physical and mental health of older adults, thereby enhancing the awareness of the risk of cognitive impairment, and helping to maintain the cognitive level of older adults. Legal aid provided by community organizations and dealing with family-neighbor relations can effectively alleviate the mental health problems of older adults, thereby preventing senile dementia to a certain extent and slowing down the decline of cognitive function.

The limitation of this study is the measurement of community service provision. The CLHLS only investigated the supply of community services, that is, whether or not older adults could access the services. However, it was uncertain whether or not older adults had used the services. Thus, further study needs to examine the association between community service use and the cognitive ability of older adults. Another limitation of this study is the problem of the causal relationship between social participation, community service provision and cognitive function. Older people with higher cognitive levels are more likely to engage in social participation and get access to community services, thereby maintaining a better cognitive level. Older people with cognitive impairment, on the other hand, may experience a certain degree of inhibition in social participation and getting access to community services, making it more difficult to improve their cognitive functions.

Nevertheless, this study was the first to examine the effects of community support services on the change of cognitive ability of Chinese older adults. This study used panel data to interpret the factors associated with the cognitive ability of older adults in China.

At the present stage, China lacks policies and relevant regulations for older adults with cognitive impairment. However, the country has not yet established a special security system for older adults with cognitive impairment. The Chinese government has not included the assessment of cognitive impairment in the public health program. In addition, public health service policies and long-term care systems do not apply to older adults with cognitive impairment. Therefore, in the future, the community should be equipped with special management for the cognitive impairment group and strengthen the early treatment and infrastructure investments appropriately. Moreover, the government should put further efforts to establish more rehabilitation training equipment and physical activity infrastructure in the community.

Furthermore, staff involved in providing health services in the community should be trained for the people with cognitive impairment so that they can get along with and care for them. The community should be equipped with professional nursing staff, general practitioners, occupational therapists, and social workers for early prevention, detection, and treatment of cognitive impairment groups.

Community health service agencies should conduct comprehensive screening of the cognitive impairment population in the community and incorporate the cognitive impairment assessment results into the health record system. They should also strengthen the popularization and publicity of cognitive impairment, popularize the knowledge of Alzheimer’s disease, carry out preventive education and lifestyle guidance for community residents, and eliminate discrimination against older adults with cognitive impairment. By doing so, people with cognitive impairment can obtain sufficient social support.

Therefore, the government should actively implement the policy of scientific and technological elderly care services, and solve various difficulties faced by older adults with cognitive impairment in obtaining community services and participating in social activities through scientific and technological innovation. Thus, older adults can better integrate into the wave of development in the era of science and technology, and protect their physical and mental health.

Therefore, government agencies should strengthen scientific and technological care for older adults, expand their scope of digital technology service facilities, provide infrastructure guarantees for older adults to participate in digital information activities, and better improve the intelligent technology operation ability of older adults. In addition, community organizations should guide and encourage the elderly to actively participate in the social interaction services provided by the Internet, stimulate their initiative in participating in online social activities, and promote older adults to better adapt to the digital society. Thus, the elderly can obtain higher-quality spiritual comfort and emotional support to effectively prevent and avoid the rapid decline of cognitive ability.

## Data Availability

The datasets generated and/or analysed during the current study are available in the CLHLS repository, https://opendata.pku.edu.cn/dataset.xhtml?persistentId=doi:10.18170/DVN/MKB1LF.

## References

[1] Alzheimer’s Disease International (2015). The global impact of dementia: An analysis of prevalence, incidence, cost and trends, Alzheimer’s Disease International (ADI).

[2] Li, P(2019). The number of dementia patients in is increasing by 300,000 annually. Qianjiang Evening News, 10-21(10). [in Chinese].

[3] Bai CH (2020). Study on the supply and effects of community health management service for urban-rural elderly under the background of pension combined with medical service: Evidence from CLHLS. Chin J Health Policy.

[4] Statistical Bureau (2021). Statistical Yearbook. Statistics Press [in Chinese].

[5] Chan, W.L.(2020). During the "13th Five-Year Plan" period, a total of 5 billion central financial funds were invested in the pilot community-services programs. CCTV Network, available at: https://m.gmw.cn/2020-10/25/content_1301719544.htm. [Accessed 28^th^ Feb 2023] [in Chinese].

[6] Duan-Porter W, Ullman K, Rosebush C, McKenzie L, Ensrud KE, Ratner E, Wilt TJ (2020). Interventions to Prevent or Delay Long-Term Nursing Home Placement for Adults with Impairments-a Systematic Review of Reviews. J Gen Intern Med.

[7] Yang F, Cao J, Qian D, Ma A (2020). Stronger Increases in Cognitive Functions among Socio-Economically Disadvantaged Older Adults in : A Longitudinal Analysis with Multiple Birth Cohorts. Int J Environ Res Public Health..

[8] Wills, T(1985). A Supportive functions of interpersonal relationships. Academic Press.

[9] Newson RS, Kemps EB (2005). General lifestyle activities as a predictor of current cognition and cognitive change in older adults: a cross-sectional and longitudinal examination. J Gerontol Series B Psychol Sci Soc Sci.

[10] Saczynski JS, Pfeifer, Masaki K (2006). The effect of social engagement on incident dementia: the Honolulu-Asia Aging Study. Am J Epidemiol.

[11] Carlson MC (2008). Exploring the facts of an everyday activity program on executive function and memory in older adults: Experience Corps. Gerontologist.

[12] James BD, Wilson RS, Barnes L, Bennett DA (2011). Late-life social activity and cognitive decline in old age. J Int Neuropsychol Soc.

[13] Cheng ST, Chan ACM, Yu ECS (2006). An exploratory study of the effect of mahjong on the cognitive functioning of persons with dementia. Int J Geriatr Psychiatry.

[14] Hsu HC (2007). Does Social Participation by the Elderly Reduce Mortality and Cognitive Impatience?. Aging Ment Health.

[15] Xue XD (2018). The effect of social participation on cognitive function of middle-aged and elderly people in my country. Popul Health.

[16] Zhu HL, Zeng XQ (2019). Social activities and the cognitive ability of rural middle-aged and old people-Evidence from CHARLS 2011–2015. Labor.

[17] Vecchio N, Fitzgerald JA, Radford K, Fisher R (2016). The association between cognitive impairment and community service use patterns in older people living in. Health Soc Care Community.

[18] World Health Organization (2015). World report on ageing and health. World Health Organization.

[19] Chaudhury H, Mahal T, Seetharaman K, Nygaard HB (2020). Community participation in activities and places among older adults with and without dementia. Dementia Int J Soc Res Pract.

[20] Yu, Y.S.H., Ma, L., Lei, J., Li, J.F., & Ma, C.H. (2018). The relationship between community support and cognitive function in the elderly: data analysis of the Chinese elderly health influencing factors tracking survey project. Chinese Mental Health Journal, 6(32 ). [in Chinese].

[21] A Catherine RN Bevil Priscilla C EdD RN O'Connor PhD Pamela M. RN, C, MSN (1994). Leisure Activity, Life Satisfaction, and Perceived Health Status in Older Adults. Gerontol Geriatr Educ.

[22] Xie QX (2019). Research on the economic burden of Alzheimer's disease and its main responsibility. Med Herald..

[23] Ma L, Tang Z, Zhang L, Sun F, Li Y, Chan P (2018). Prevalence of Frailty and Associated Factors in the Community-Dwelling Population of. J Am Geriatr Soc.

[24] Chiu HF, Lee HC, Chung WS, Kwong PK. Reliability and validity of the Cantonese version of mini-mental state examination—A preliminary study. Hong Kong Journal of Psychiatry. 1994;4:25.

[25] Murayama H, Sugiyama M, Inagaki H, Okamura T, Miyamae F, Ura C, Awata S (2018). Is community social capital associated with subjective symptoms of dementia among older people? A cross-sectional study in. Geriatr Gerontol Int.

[26] Tang FY, Zhang W, Chi I, Li MT, Dong XQ (2020). Importance of Activity Engagement and Neighborhood to Cognitive Function Among Older Chinese Americans. Res Aging.

[27] Zhang RH, Su Q (2019). The impact of community home-based eldelycare services on the health of the elderly: an empirical analysis from CLHLS data. Aging Sci Res.

[28] Wei M, Wang JW (2021). The Impact of Rich Community Environment on the Cognitive Trajectory of Urban Elderly. Popul Dev.

[29] Yu YS (2021). Longitudinal association between home and community-based services provision and cognitive function in Chinese older adults: Evidence from the Chinese Longitudinal Healthy Longevity Survey. Health Soc Care Commun.

